# Small Bowel Perforation in Roux-en-Y Gastric Bypass (RYGB) Secondary to Apolipoprotein A-IV (AApoA-IV) Type Amyloidosis

**DOI:** 10.1007/s11695-025-07814-8

**Published:** 2025-03-29

**Authors:** Mona Zhi Ling Mai Jiang, Stefaan De Clercq

**Affiliations:** 1 St Vincent’s Hospital, Melbourne, Australia; 2 Friendly Society Private Hospital, Bundaberg, Australia

**Keywords:** Amyloidosis, Crohn's Disease, Ulcerative Colitis, Inflammatory Bowel Disease, Bariatric Surgery

## Abstract

**Background:**

ApoA‐IV amyloidosis is a rare disease that involves the deposition of ApoA-IV protein aggregates in tissues. It commonly presents as cardiac or renal disease, but can, in rare cases, cause small bowel perforation.

**Methods:**

This study describes a case of ApoA-IV type amyloidosis causing small bowel perforation after conversion of a sleeve gastrectomy (SG) into a RYGB in a Crohn’s disease (CD) and rheumatoid arthritis (RA) patient. It also considers the indications for bariatric and anti-reflux surgery in the setting of co-morbid inflammatory bowel disease (IBD), gastro-oesophageal reflux disease (GORD), and obesity.

**Results:**

Obesity can reduce the efficacy of IBD medications and drives a pro-inflammatory state that may worsen IBD, however IBD patients present an operative challenge due to risk of more intestinal adhesions, potential intolerance to intestinal bypass, and risk of affecting options for future bowel resections if required. SG is often chosen over RYGB for CD patients due to limited short-term complications. However, when considering co-morbid GORD, the long-term risk of medication resistant GORD, erosive oesophagitis, and Barrett’s oesophagus with SG is significant, especially given that SG is an irreversible procedure.

**Conclusion:**

There is growing evidence that bariatric surgery in IBD patients is both safe and effective, however the decision to perform bariatric surgery in an IBD patient involves consideration of the intricate interplay between obesity and IBD.

## Background

Amyloidosis is a heterogenous group of diseases involving abnormal protein deposition. Though rare, one must consider amyloidosis as a potential cause of small bowel perforation in patients with IBD. There is an increasing prevalence of obesity among IBD patients, estimated at approximately 15–30%, as well as a growing number of bariatric operations being performed [1–5]. Bariatric surgery is increasingly being offered to patients with co-morbid obesity and IBD, due to mounting evidence of its safety and efficacy in this cohort [4, 6–11]. Though bearing significant potential benefit, the decision to perform bariatric surgery in IBD patients is a balance of multiple factors. This study describes a rare case of apolipoprotein A-IV (AApoA-IV) type amyloidosis causing small bowel perforation post-RYGB and examines the considerations involved when choosing to perform bariatric surgery on an IBD patient.

## Case Presentation

A woman in her 50s presented approximately 2 years post-SG with a hiatal hernia and severe mixed acid and biliopancreatic reflux. Her symptoms, including progressively worsening nausea, burping, and burning epigastric pain, not controlled by proton pump inhibitors and anti-emetics, resulted in extremely poor quality of life. Her past medical history was significant for CD, first diagnosed in 2022 and managed with adalimumab, as well as RA, diagnosed approximately in the 1980s and managed with methotrexate and non-steroidal anti-inflammatories. Other medical history included GORD, iron deficiency anaemia, laparoscopic cholecystectomy, caesarean section, and previous bilateral total hip replacements with multiple revisions for a delayed diagnosis of hip dysplasia.

SG was initially chosen in order to prevent short-term, medication-associated complications related to RYGB, as was commonly suggested in the literature at that time. However, recent literature demonstrates that complication rates after various weight loss surgeries are comparable between IBD and non-IBD patients (see ‘Discussion’ for further details). Given the absence of a fundus, a hiatal hernia repair with fundoplication was not possible. Therefore, a decision was made to convert her SG to a RYGB. The preoperative screening upper and lower endoscopies demonstrated a 4 cm mixed hiatal hernia, bilious fluid in the stomach, gastritis, mild diverticulosis, and some small hyperplastic colonic polyps. Coeliac disease, disaccharidase deficiency, endocrine disorders, *Helicobacter pylori* infection, and psychological contraindications were excluded pre-operatively. Adalimumab was discontinued perioperatively.

**Fig. 1:**
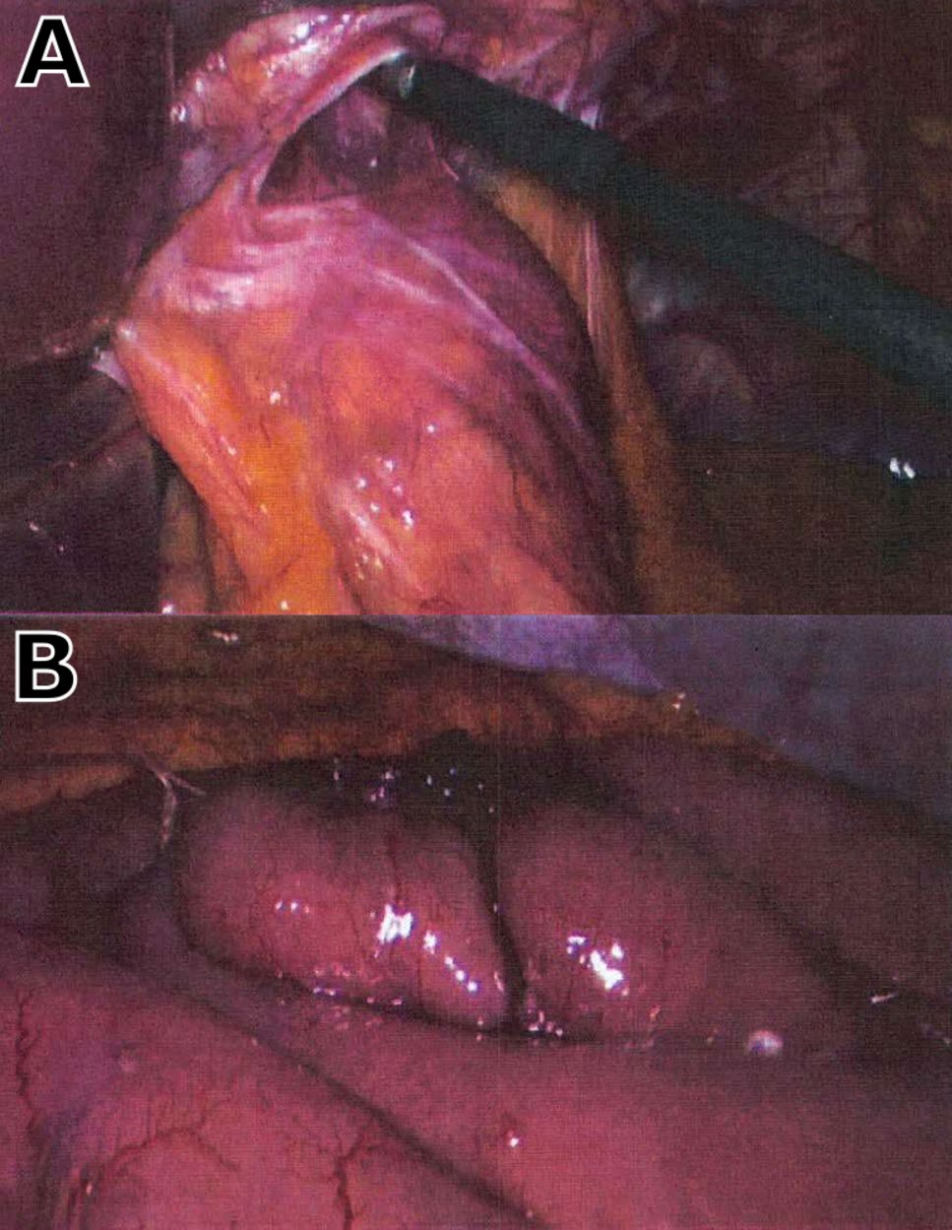
Intraoperative images from conversion of SG to RYGB. **A** Hiatal hernia. **B** Distal RYGB anastomosis

Intraoperatively, a 3.5 m common limb was designed, as opposed to the usual 3 m, to accommodate a possible future resection of the terminal ileum (see Fig. 1). The perioperative course of this conversion was unremarkable, and a short segment of resected small bowel, from in between the two anastomoses, was confirmed to be viable with no histological abnormalities. The patient was discharged on day 3 post-operatively and was progressing well on review in the surgical clinic, 1 week post-operatively.

She represented to the emergency department 15 days post-operatively, with progressively worsening epigastric and left-sided colicky abdominal pain, nausea and vomiting. Her temperature was 36.4 °C, and her observations were within normal limits. She had epigastric and left upper quadrant tenderness on palpation, without signs of guarding, rigidity, or peritonism. She had a neutrophilia of 11.65 × 10⁹/L with a total white cell count of 14.3 × 10⁹/L. There was an associated elevated platelet count of 460 × 10⁹/L and elevated C-reactive protein of 44 mg/L. Her routine blood biochemistry was otherwise unremarkable.

**Fig. 2:**
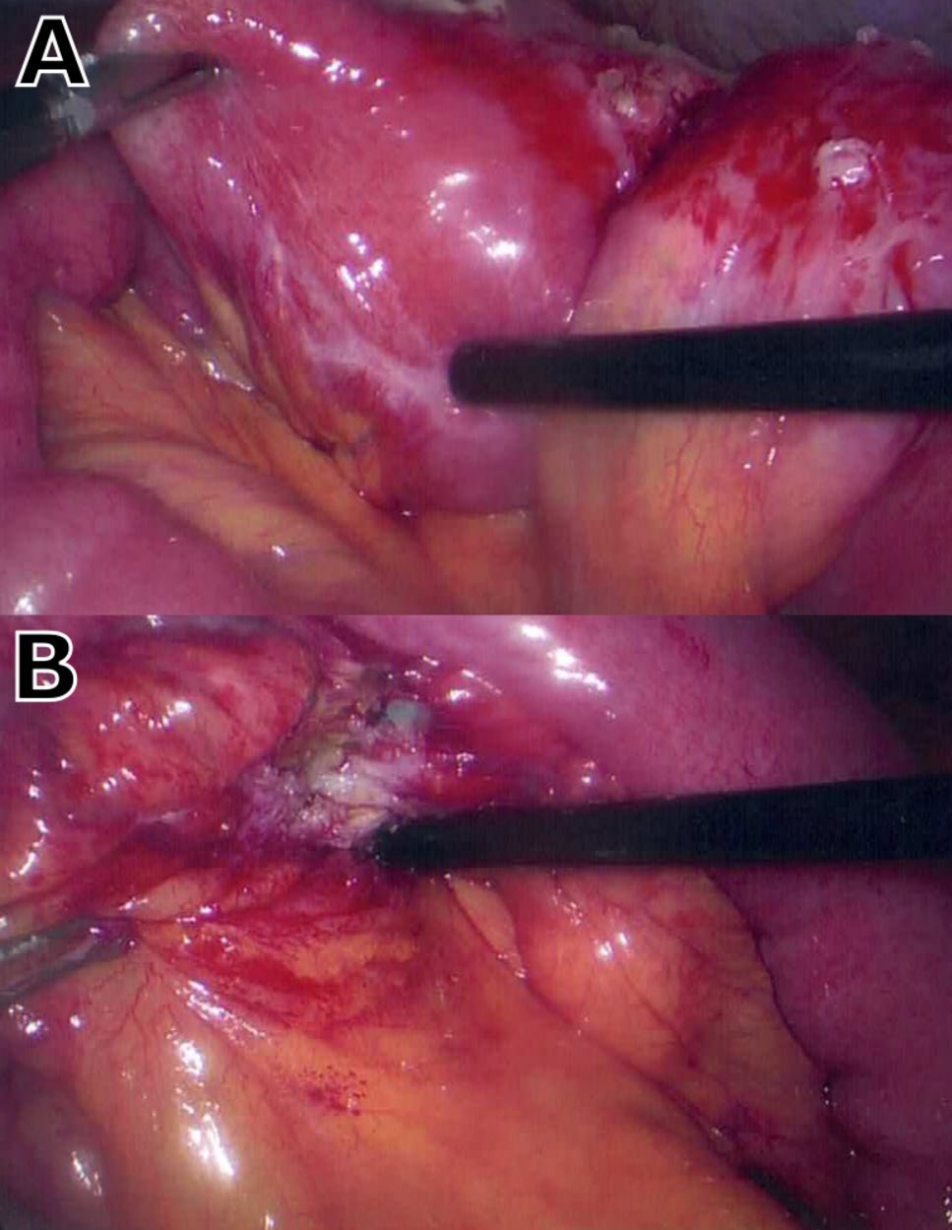
Intraoperative images from revision of RYGB, with resection of contained perforation and formation of jejunojejunostomy. **A** Distal RYGB anastomosis. **B** Exposed small bowel perforation

Computed tomography (CT) abdomen and pelvis showed distension of jejunal bowel loops in the left upper quadrant, with a transition point near the distal anastomotic site. She was admitted to hospital with a presumed diagnosis of adhesional small bowel obstruction and returned to theatre for laparoscopic adhesiolysis and revision of the distal anastomosis. Intra-operatively, a limited, contained small bowel perforation was noted at the distal anastomosis, with surrounding dense adhesions. The initial distal anastomosis was resected and sent for histopathology, with a new distal anastomosis formed via jejuno-jejunostomy (see Fig. 2). Her post-operative recovery was unremarkable, and she was discharged on day 5 after the revision operation.

**Fig. 3:**
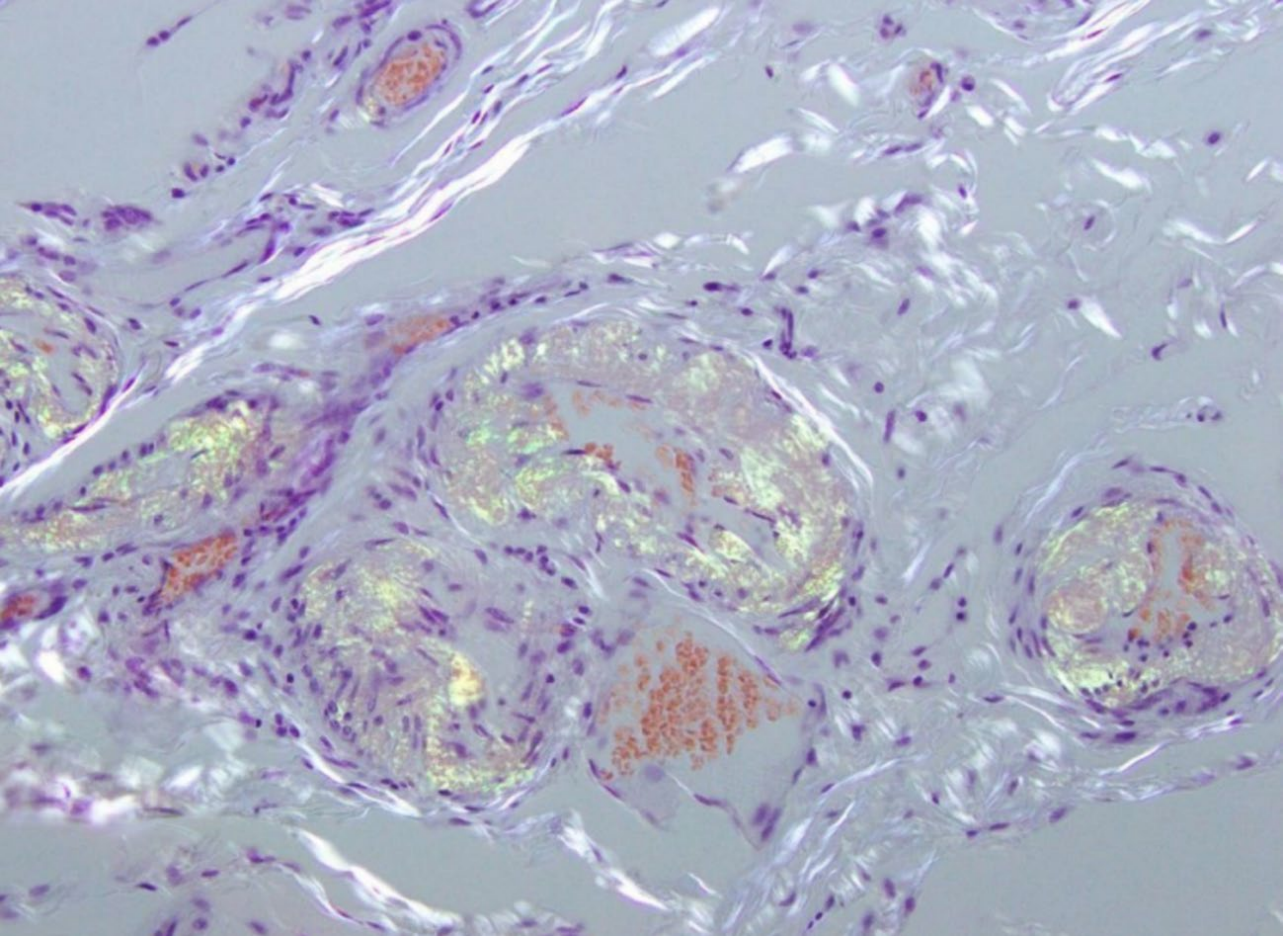
A representative section of small bowel wall, showing green birefringence on polarisation (Congo red), consistent with amyloidosis

On histopathological analysis, the small bowel mucosa showed a site of perforation and was otherwise macroscopically unremarkable. Microscopically, the resected margins were viable. There was congophilia of the small arteries and arterioles of the submucosa, with apple green birefringence on polarisation of the slide (Congo red stain). There was metachromasia on crystal violet stain (see Fig. 3). These microscopic changes were consistent with amyloidosis, and further amyloid subtyping via liquid chromatography–mass spectrometry favoured AApoAIV type amyloidosis.

She was reviewed in the surgical clinic 2 weeks after the revision operation and was recovering well. She had follow-up in the amyloidosis clinic, with no evidence of the more common cardiac or renal amyloid complications on screening investigations. On phone review 16 months after the revision operation, she described minimal reflux-related symptoms; she was able to return to her usual work and personal duties, but continues to have persistent fatigue.

## Discussion

Amyloidosis comprises a group of heterogeneous diseases, involving deposition of protein aggregates in various tissues [12]. It is subdivided by type according to the pathogenic fibril precursor protein [13]. Reactive or serum amyloid A protein (AA) amyloidosis is a common form of amyloidosis and occurs secondary to elevated serum amyloid A protein, which is seen in chronic inflammatory states [14]. Its synthesis in hepatocytes is regulated primarily by IL-1 and IL-6, with SAA levels increasing in response to inflammation and remaining elevated until the inflammation resolves [13]. In this case, our patient had CD and RA, both of which can cause AA amyloidosis, although notably, this was not the amyloidogenic protein in question. Systemic AA amyloidosis secondary to IBD has an estimated prevalence of 0.53% and occurs more commonly in CD than ulcerative colitis (UC) [13, 15, 16]. This may be explained by the more pronounced acute phase response seen in CD, which may be related to its higher prevalence of suppurative features [13]. Additionally, surgical resection can act as definitive management for UC, leading to improved long-term control of systemic inflammation, whereas the same is not possible in CD [13]. However, systemic AA amyloidosis in the setting of IBD commonly presents as renal and cardiac disease, as opposed to gastrointestinally [17].

AA amyloidosis can be treated through control of the underlying inflammatory process. A case study by Prayman *et al.* noted renal impairment or proteinuria in all included patients, with an association between poorly controlled IBD, raised inflammatory markers, and progressive renal impairment [13]. Consequently, reduced inflammation and lowered production of SAA can also lead to amyloid resorption via macrophage-related activity, although the exact mechanism is unclear [13]. In this case, the patient was on adalimumab, which was ceased pre-operatively. Adalimumab is a humanised immunoglobulin G1 monoclonal antibody directed against tumour necrosis factor-alpha (anti-TNF-alpha) [18]. A study by Kuroda *et al.* showed a reduction in gastroduodenal AA amyloid deposits upon successful treatment with anti-TNF-alpha therapy, in patients with rheumatoid arthritis [19]. However, this was not the amyloidogenic protein found here.

This case demonstrated gastrointestinal ApoA-IV type amyloidosis, a rare and under-investigated form of amyloidosis. ApoA-IV is a 46kD protein that is secreted from the small bowel in response to lipid absorption and chylomicron formation; however, its specific biological role has yet to be identified [20, 21]. It may have antioxidant, anti-inflammatory and atheroprotective functions [22]. Plasma levels of ApoA-IV increase with age; however, it is unclear if high levels of ApoA-IV leads to ApoA-IV amyloidosis [23, 24]. Analysis of patients attending the UK NHS National Amyloidosis Centre revealed 15 cases of ApoA‐IV amyloidosis, with only a singular case of duodenal ApoA‐IV amyloidosis [25]. The Mayo Clinic’s review of their mass spectrometry database indicated that 0.45% of all 9673 cases of amyloidosis from all organs were ApoA‐IV amyloidosis [24]. In our search of the literature, no other documented cases of jejunal ApoA‐IV amyloidosis were found. ApoA‐IV amyloidosis is typically systemic, with renal and cardiac manifestations being the most common [26]. Renal findings include progressively declining renal function with minimal proteinuria [24]. Renal biopsies showed amyloid deposits in the renal medulla for all cases, with some cases also having peritubular amyloid deposits; the renal cortex was spared, and there was no interstitial inflammation [24, 27]. Cardiac manifestations of ApoA‐IV amyloidosis included left ventricular outflow tract obstruction and coronary artery disease [28]. Histopathological samples of cardiac tissue showed obstructive microvascular amyloidosis in all cases and nodular interstitial deposition in some cases [28]. Unlike other forms of amyloidosis, ApoA‐IV amyloidosis has no specific treatments [24].

In this case study, SG was the initially chosen bariatric procedure, however the patient developed medication resistant GORD, necessitating conversion to RYGB. SG is the most commonly performed bariatric surgery [5]. It is especially favoured due to the short operative time and relative technical simplicity, which is advantageous in patients with significant respiratory or cardiovascular comorbidities, or those with extensive adhesions or a restricted operative field due to extremely high BMI [29]. SG can also be performed in patients where intestinal bypass is a relative contraindication, such as those on anti-inflammatory medications or patients with IBD [29]. However, SG is irreversible and is less effective than RYGB for sustained weight loss and remission of obesity-associated comorbidities [29, 30]. Additionally, the effect of SGs on GORD is variable; some studies show improvement in GORD, likely secondary to weight loss and reduced gastric acid secretion, while others indicate worsening GORD or de novo GORD post-SG [29, 31]. A meta-analysis by Yeung *et al.* has estimated rates of de novo GORD post-SG at 23%, oesophagitis at 28%, and Barrett’s oesophagus (BE) at 6% [32]. A prospective randomised study by Genco *et al.* demonstrated that the cumulative incidence of oesophagitis post-SG was up to 74.7% 5 years post-operative, often with associated biliopancreatic reflux, generating BE with an incidence of 8% [29]. Severe medication-resistant GORD is the most common indication for revision surgery post-SG [32]. Additionally, SG eliminates the possibility of fundoplication should severe GORD occur post-operatively. Therefore, RYGB is still commonly considered the gold standard for management of co-morbid obesity and GORD [31].

The decision to perform bariatric and anti-reflux surgery in IBD patients is a complex one, taking into account the risk of operative complications, the risks of long-term GORD, and the benefit of weight loss on both general health as well as the clinical course of IBD. There is evidence that bariatric surgery is both safe and effective in IBD patients, with numerous studies showing no difference in weight loss, quality of life, or postoperative complications between IBD and non-IBD cohorts [6, 7, 9–11, 33]. Some studies noted some variations on subgroup analysis. A systematic review by Aziz *et al.* found that post-operative bleeding and wound infections occurred at higher rates in IBD patients [10]. In a cohort study by Wallhuss *et al.*, higher rates of postoperative complication were noted in RYGB patients with UC, and higher re-admission rates were noted among CD patients; weight loss was found to be marginally better with RYGB in IBD patients [7]. Additionally, in the setting of comorbid IBD and obesity, successful weight loss has been associated with fewer IBD-related complications [8]. This may be related to the reduced efficacy of IBD medications in the setting of obesity [34, 35]. A case series by Aminian *et al.* noted improvement in IBD symptoms in 9 of 10 patients post weight loss due to bariatric surgery [4]. This may be due to the pro-inflammatory nature of obesity, with over-expression of IL-6, TNF-alpha, and adipokines in visceral and mesenteric fat [4]. It follows that weight loss may reduce the production of obesity-related pro-inflammatory cytokines and therefore reduce IBD disease activity [4]. A meta-analysis by Garg *et al*. noted that only 11% of patients had increasing IBD medication requirements post-bariatric surgery, with the majority experiencing no change, and 46% experiencing a reduction in IBD medication [9].

## Data Availability

No datasets were generated or analysed during the current study.
